# Mississippi Valley Dental Society Meeting

**Published:** 1872-03

**Authors:** 


### M. V. D. S. MEETING.

The grand old Mississippi Valley Dental Society has just closed one of its best annual meetings, from the 6th to the 8th inst. (March) inclusive. It was one of the best, if not the very best meeting this Society has ever held; and that is saying a great deal; for it is perhaps due to say that for interest and instruction, the annual meetings of this body have no superior, if an equal, anywhere.

The subjects presented were either new, or in a new dress, and so it was necessary to introduce some of the members to them, for they really did not seem to know whether they had ever met them before or not. Others thought they had, but not with those clothes on. We are so much in the habit of judging by costume that sometimes we don't know our most intimate friends when they are "dressed up." But in this case an acquaintance was soon formed, and all went to work with a good will. Some to talking, but a far larger number to listening: An observation in all dental meetings, would lead any one to the conclusion that there are far more listeners than talkers, and perhaps upon the whole, this is about right, for a great many can listen who will not talk; and what would a speech be without an audience.

A larger number were in attendance than ever before, a few of these, however, had an additional attraction in another matter, viz: to learn if somebody couldn't help them out of trouble, or save them from their "Bacon." Almost everybody wishes to have his Bacon saved, especially if it is in danger; but there is a certain class just now who are frantically in earnest to be saved *from* their "Bacon." They have got a "Bacon" and they don't know what to do with him; they want to salt him, or have him salted, but he objects, and insists on salting other people, and then rub(ber)ing it in, about forty dollars worth annually. Well now really, this thing of having the rubbers put on hard isn't agreeable, and so we had a convention of the representative men of the profession of the West, to see what should be done about it, and after mature deliberation, it was agreed to thank somebody—we forget who—for something—we forget what. They also agreed to do something, and we forget what that was, or when it should be done, or who should do it. There was an energetic and inopportune effort made to have the M. V. D. S. do it, but it would not. It is quite apparent that we can't tell what was done at that convention, and so we will refer the reader to its proceedings on the opposite page of this number.

